# Hypoxia-inducible factor 1–mediated characteristic features of cancer cells for tumor radioresistance

**DOI:** 10.1093/jrr/rrw012

**Published:** 2016-08-16

**Authors:** Hiroshi Harada

**Affiliations:** 1 Department of Radiation Oncology and Image-applied Therapy, Kyoto University Graduate School of Medicine, 54 Shogoin Kawahara-cho, Sakyo-ku, Kyoto 606-8507, Japan; 2 Hakubi Center, Kyoto University, Yoshida-Honmachi, Sakyo-ku, Kyoto 606-8501, Japan; 3 Precursory Research for Embryonic Science and Technology (PRESTO), Japan Science and Technology Agency (JST), 4-1-8 Honcho, Kawaguchi, Saitama 332-0012, Japan

**Keywords:** hypoxia-inducible factor 1 (HIF-1), radioresistance, cancer, metabolic reprogramming, tumor blood vessels, cell cycle, Warburg effect

## Abstract

Tumor hypoxia has been attracting increasing attention in the fields of radiation biology and oncology since Thomlinson and Gray detected hypoxic cells in malignant solid tumors and showed that they exert a negative impact on the outcome of radiation therapy. This unfavorable influence has, at least partly, been attributed to cancer cells acquiring a radioresistant phenotype through the activation of the transcription factor, hypoxia-inducible factor 1 (HIF-1). On the other hand, accumulating evidence has recently revealed that, even though HIF-1 is recognized as an important regulator of cellular adaptive responses to hypoxia, it may not become active and induce tumor radioresistance under hypoxic conditions only. The mechanisms by which HIF-1 is activated in cancer cells not only under hypoxic conditions, but also under normoxic conditions, through cancer-specific genetic alterations and the resultant imbalance in intermediate metabolites have been summarized herein. The relevance of the HIF-1–mediated characteristic features of cancer cells, such as the production of antioxidants through reprogramming of the glucose metabolic pathway and cell cycle regulation, for tumor radioresistance has also been reviewed.

## INTRODUCTION

High-precision radiation therapy enables radiation oncologists to decrease delivery of an excessive dose of radiation to normal tissues and also to administer a high and booster dose of radiation, particularly to small target fractions in a malignant tumor [1]. In order to efficiently use this technique in the future for cancer patients, a better understanding of the characteristics of radioresistant cancer cells in malignant solid tumors is needed. Based on accumulating evidence, molecular oxygen is recognized as one of the most influential factors on the cytotoxic effects of radiation; in other words, cancer cells may be radioresistant under hypoxic conditions in solid tumors [2–4]. This has been attributed to the reactivity of molecular oxygen, e.g. oxygen has high affinity for free radicals produced by radiation in cellular components such as DNA and maintains them in unrepairable forms [2, 3]. In addition to this chemical mechanism of radiation, cellular adaptive responses to hypoxia mediated by the hypoxia-inducible transcription factor, HIF-1, have been suggested to function in the induction of biological radioresistance in cancer cells under hypoxic conditions; therefore, HIF-1 has been attracting increasing attention in the field of radiation oncology [3–7].

On the other hand, biochemistry and molecular biology–based research on the molecular mechanisms underlying the activation of HIF-1 have revealed that the deprivation of oxygen is not necessarily important [8, 9]. For example, some genetic alterations have been shown to lead to the activation of HIF-1, even in the presence of oxygen, through decreases in the levels of either Fe^2+^ or an intermediate metabolite of the tricarboxylic acid cycle (TCA cycle), α-ketoglutarate [8, 9]. Since HIF-1 is strongly associated with the radioresistant phenotypes of cancer cells, conditions that potentially lead to the activation of HIF-1 are now recognized as playing crucial roles in tumor radioresistance.

The molecular mechanisms responsible for the activation of HIF-1 have been described herein, and the functions of HIF-1 in the induction of tumor radioresistance and tumor recurrence after radiation therapy have been reviewed.

## MECHANISMS UNDERLYING THE ACTIVATION OF HIF-1

### Activation of HIF-1 under hypoxic conditions

**Fig. 1.**
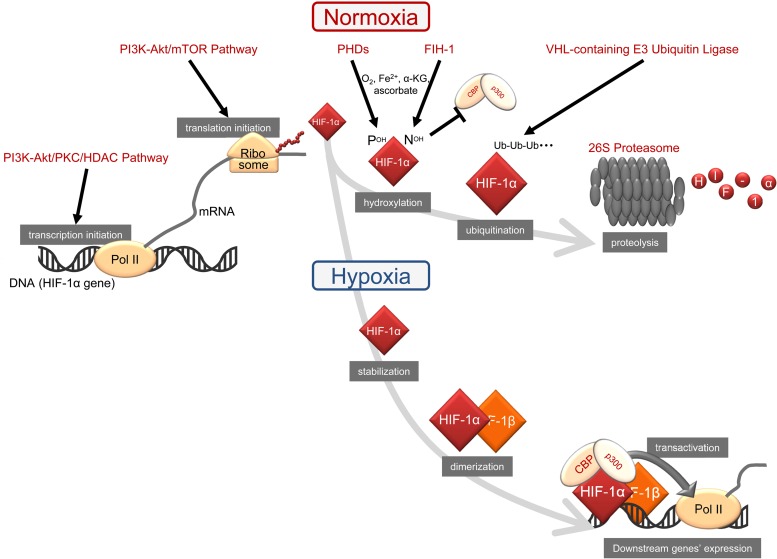
Molecular mechanisms underlying the activation of HIF-1.

HIF-1, which was originally identified as a transcription factor for the expression of the erythropoietin (*EPO*) gene under hypoxic conditions [10–12], is composed of alpha and beta subunits, HIF-1α and HIF-1β, respectively (Fig. 1). In contrast to the constitutive expression of HIF-1β, the expression levels and transactivation activity of HIF-1α are downregulated in an oxygen-dependent manner, which is mainly responsible for the hypoxia-dependent activation of the heterodimer, HIF-1 [13]. More specifically, the HIF-1α protein is hydroxylated on two proline residues (P402 and P564) and one asparagine (N803) residue by two kinds of dioxygenases, prolyl hydroxylases (prolyl-4-hydroxylases: PHDs) and asparaginyl hydroxylase (factor-inhibiting HIF-1: FIH-1), respectively [13]. These dioxygenases require molecular oxygen for their activity and, thus, do not function under oxygen-deprived conditions [13]. Oxygen-dependent and PHDs-mediated prolyl hydroxylation lead to the ubiquitination of HIF-1α by von Hippel Lindau (VHL)-containing E3 ubiquitin ligase and subsequent proteolysis by the 26S proteasome, which is responsible for the oxygen-dependent degradation of the HIF-1α protein [13–18]. Oxygen-dependent and FIH-1–mediated asparaginyl hydroxylation suppress the recruitment of the transcriptional co-activators, p300/CREB-binding protein (CBP) acetyltransferases, to the HIF-1α protein, resulting in the oxygen-dependent blocking of the transactivation activity of the HIF-1α protein [13, 19]. In contrast, the HIF-1α protein becomes stable and acquires transactivation activity under hypoxic conditions because of the inactivation of these oxygen-dependent dioxygenases [13]. HIF-1α then interacts with its binding partner HIF-1β, and the resultant heterodimer, HIF-1, begins to induce the transcription of no less than 800 genes that function in adapting to hypoxic environments (e.g*.* metabolic reprogramming [20–23]), escaping hypoxic conditions (invasion and metastasis of cancer cells [24–26]), and improving oxygen availability (angiogenesis and neovascularization [7, 23, 25]).

### Hypoxia-independent activation of HIF-1

In recent years, studies have demonstrated that HIF-1 becomes active even in the presence of oxygen when the following conditions are satisfied.

#### Decrease in activity of dioxygenases

Since the hydroxylation activity of dioxygenases is dependent not only on oxygen, but also on Fe^2+^ and α-ketoglutarate, HIF-1 becomes active when the levels of either of these decreases in cells, even in the presence of oxygen [27, 28]. A dysfunction in the TCA cycle has been associated with reductions in α-ketoglutarate levels and the resultant activation of HIF-1. For example, Selak *et al*. reported that a deficiency in succinate dehydrogenase (SDH), which is often confirmed in some types of cancers, results in the accumulation of succinate in cells [29]. Since succinate potentially inhibits the decarboxylation of α-ketoglutarate to succinate and increases the intracellular levels of α-ketoglutarate, a SDH deficiency in cancer cells inhibits both prolyl and asparaginyl hydroxylases, thereby upregulating the stability and transactivation activity of HIF-1α [29]. Zeng *et al*., from my laboratory, showed the importance of abnormalities in the expression of another TCA cycle enzyme in the reduction of α-ketoglutarate levels [23]. We found that the aberrant expression of the wild-type isocitrate dehydrogenase 3α gene (*IDH3α*) reduced α-ketoglutarate levels and increased the stability and transactivation activity of HIF-1α by facilitating the reductive carboxylation of α-ketoglutarate to isocitrate in transformed cells [23]. In addition to decreased α-ketoglutarate levels, Isaacs *et al*. revealed that fumarate, the levels of which are elevated as a result of a fumarate hydroxylase deficiency in cancer cells, also increases the expression levels of HIF-1α through inhibition of the activity of PDHs by directly competing with its co-factor, α-ketoglutarate [30].

Factors that potentially decrease intracellular Fe^2+^ levels are also influential in the activation of HIF-1 activity under normoxic conditions because Fe^2+^ is an essential co-factor for PHDs and FIH-1 as described above [27, 28]. One example is the influence of reactive oxygen species (ROS). ROS have been shown to play pivotal roles as messengers in signal transduction and cell cycle regulation [31]. Since one of the main sources of ROS is mitochondria, dysfunctional mitochondria in cancer cells produce excessive amounts of ROS, leading to decreases in Fe^2+^ levels through its oxidation to Fe^3+^. Thus, the aberrant accumulation of ROS induces HIF-1 activity by inactivating dioxygenases [28]. Although ionizing radiation also induces the production of ROS and HIF-1 activity, its details will be described in a later section on tumor radioresistance (see Section 4.1).

#### Impairments in E3 ubiquitin ligase and deubiquitination

After the hydroxylation of P402 and P564 in HIF-1α, VHL-containing E3 ubiquitin ligase recognizes the HIF-1α protein for ubiquitination, leading to the proteolysis of HIF-1α through the ubiquitin–proteasome system [13–18]. Therefore, a VHL deficiency is also considered to cause the accumulation of the HIF-1α protein, even in the presence of oxygen [27]. Park *et al*. highlighted the importance of mutations in another E3 ligase component, Cullin 2 (*CUL2*) [32]. They identified *CUL2* frameshift mutations in colon cancers and suggested that mutations inactivate the tumor suppressor function of *CUL2* through HIF-1α proteolysis [32].

Goto *et al*., from my lab, recently demonstrated the critical role of a deubiquitinating enzyme in the stabilization of HIF-1α [24]. In a genetic screening experiment, we identified ubiquitin C-terminal hydrolase L1 (UCHL1) as a novel deubiquitinating enzyme for the HIF-1α protein [24]. The aberrant overexpression of UCHL1 stabilized the HIF-1α protein and induced HIF-1 activity under normoxic and hypoxic conditions [24].

#### Increases in transcription and translation initiation of HIF-1α

In addition to post-translational mechanisms, regulation at the level of transcription and translation initiation is also important for the activation of HIF-1 under normoxic conditions (Fig. 1). Koshikawa *et al*. reported that ROS generated in mitochondria upregulated the transcription of the *HIF-1α* gene *via* the phosphatidylinositol 3 kinase-Akt/protein kinase C/histone deacetylase (PI3K-Akt/PKC/HDAC) pathway, leading to the accumulation and activation of HIF-1α in tumor cells [33]. Regarding translational initiation of the *HIF-1α* gene, Laughner *et al*. demonstrated the importance of signal transduction cascades [34]. Their *in vitro* study revealed that HER2 signaling did not affect the half-life of the HIF-1α protein, but stimulated its synthesis in a PI3K/Akt/mammalian target of rapamycin (mTOR)-dependent manner [34].

## HIF-1-MEDIATED RADIORESISTANCE OF CANCER CELLS

Recent advances in molecular biological research in the fields of radiation oncology and biology have led to the potential of HIF-1 in enhancing the radioresistance of cancer cells being recognized [5, 7, 35]. The functions of HIF-1 in the induction of tumor radioresistance through the reprogramming of glucose metabolism and cell cycle regulation have been summarized.

### Reprogramming of glucose metabolism and resultant overproduction of antioxidants

**Fig. 2.**
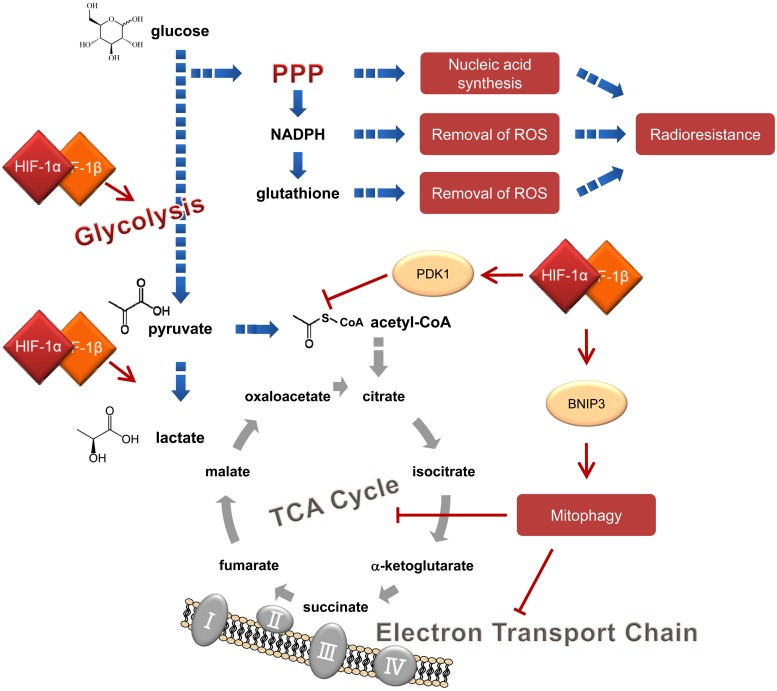
HIF-1–mediated reprogramming of the glucose metabolic pathway from the mitochondrial oxidative phosphorylation to glycolysis, which is the so-called ‘Warburg effect’. The reprogramming coupled with the activation of the pentose phosphate pathway (PPP) induces radioresistance of cancer cells.

The growth advantage of cancer cells has been attributed to unique glucose metabolic pathways that produce ATP through accelerated glycolysis rather than mitochondrial oxidative phosphorylation under normoxic as well as hypoxic conditions. This is known as the Warburg Effect (Fig. 2) [36, 37]. The transcription factor, HIF-1, plays fundamental roles in the reprogramming as followings [9, 22, 36–38]. HIF-1 accelerates glycolysis by inducing the expression of glycolytic enzymes, except for phosphoglyceric acidmutase (PGAM), at the transcription level [22, 39]. Moreover, HIF-1 inhibits the conversion of pyruvate to a substrate of the TCA cycle, acetyl-CoA, by inducing the expression of pyruvate dehydrogenase kinase 1 (PDK1) [40]. Furthermore, HIF-1 induces mitochondrial autophagy, mitophagy, by upregulating the expression of BCL2/adenovirus E1B 19 kDa interacting protein 3 (BNIP3) in order to directly reduce the mitochondrial mass [21, 41]. The HIF-1-mediated metabolic reprogramming, especially increased expression of glycolytic enzymes, e.g. glucose transporter-1 (GLUT1) and hexokinase 2 (HK2), leads to the increase in intracellular levels of glucose and glucose-6-phosphate. Since glucose-6-phosphate serves as a substrate for the pentose phosphate pathway (PPP) responsible for the biogenesis of the antioxidants NADPH and glutathione [42], the HIF-1–mediated Warburg effect has been associated with the induction of antioxidant capacity and eventual radioresistance of cancer cells (Fig. 2) [43]. We actually confirmed that accelerated glycolysis mediated by aberrant overexpression of IDH3α, and the resultant upregulation of HIF-1 activity led to the radioresistance of cancer cells. However, the molecular mechanism underlying the relationship between the HIF-1–mediated metabolic reprogramming and PPP has not necessarily been elucidated yet. For example, although functional and mechanistic links between HIF-1 and p53 have been reported so far, it remains unclear whether they function in balancing the metabolic reprogramming and the PPP. In addition, whereas it has been reported that p53 potentially suppresses the PPP by inactivating a rate-limiting enzyme of PPP, glucose-6-phosphate dehydrogenase (G6PD) [44], p53 was also demonstrated to induce the expression of TP53-induced glycolysis and apoptosis regulator (TIGAR), which promotes the PPP through the activation of G6PD and functions in the maintenance of redox homeostasis and radioresistance of cells [42, 43]. A hint to accessing these missing links may be in the line of a recent report that TIGAR activates HK2 through their direct interaction under hypoxic conditions in a HIF-1–dependent manner [45]. Also, as suggested by Stanton, the balance of G6PD stimulatory versus G6PD inhibitory signals downstream of p53 may be important in the redox homeostasis and resultant radioresistance of cancer cells [46].

### Cell cycle regulation

The Bergonie–Tribondeau's law in the field of radiation oncology and biology indicates that cells are generally more sensitive to radiation when they exhibit a marked increase in proliferative capacity [47, 48]. Therefore, HIF-1–mediated cell cycle arrest or retardation has been suggested to play a role in enhancing the radioresistance of cancer cells. Several independent studies recently demonstrated that cell cycle progression was blocked at G_1_/S transition under hypoxic conditions. Koshiji *et al*. showed that hypoxia induced G_1_ cell cycle arrest by upregulating the expression of CDNK1A, which encodes the CDK2 inhibitor p21^Cip1^, through the displacement of Myc from the CDNK1A promoter via HIF-1α [49]. Hammer *et al*., from the same group, also demonstrated that hypoxia inhibited the expression of CDC25A, another cell cycle regulator encoding a tyrosine phosphatase that maintains CDK2 activity, resulting in cell cycle arrest at the G_1_ checkpoint [50]. Although cells are more radiosensitive at the late G_1_ and early S phases than at the late S phase, cell cycle arrest/retardation at any checkpoint is considered to enhance the radioresistance of cancer cells, based on the Bergonie–Tribondeau's law.

## HIF-1-MEDIATED PROMOTION OF TUMOR RECURRENCE AFTER RADIATION THERAPY

In addition to the function of HIF-1 in the induction of the radioresistant characteristics of cancer cells, other mechanisms, by which tumor recurrence after radiation therapy is promoted as a result of the activation of HIF-1, have recently been proposed.

### Protection of tumor blood vessels

**Fig. 3.**
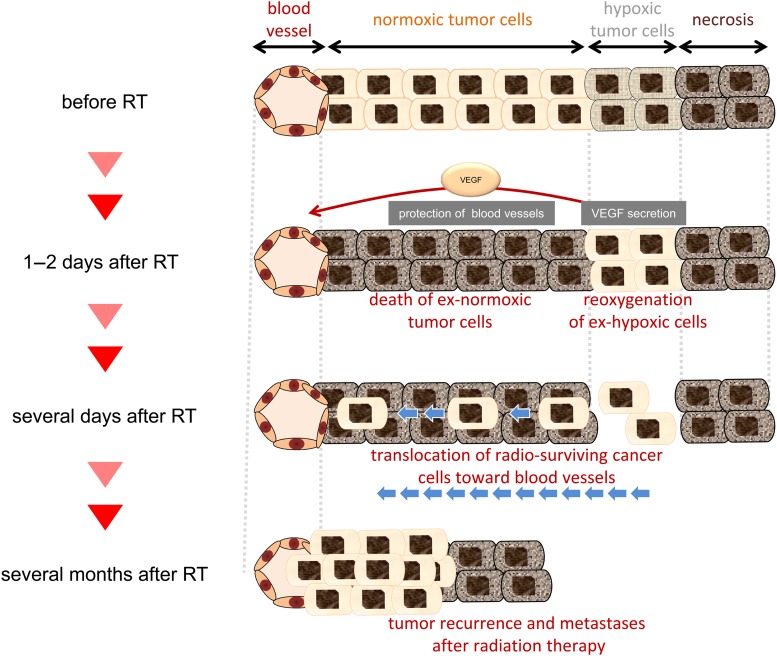
Dynamics of cancer cells after radiation therapy. Hypoxic tumor cells predominantly survive radiation therapy and protect blood vessels by secreting VEGF in a HIF-1–dependent manner. The radiosurviving ex-hypoxic cancer cells translocate toward the blood vessels and cause tumor recurrence and distant tumor metastases after radiation therapy [6].

Radiation therapy exerts cytotoxic effects not only on cancer cells, but also on the tumor vasculature in its treatment field. Previous studies suggested the existence of a phenomenon in which cancer cells protect the tumor vasculature from the cytotoxic effects of radiation in a HIF-1–dependent manner, as follows (Fig. 3) [5, 51, 52]. Radiation efficiently kills cancer cells in normoxic regions, resulting in the so-called reoxygenation of hypoxic tumor cells [51, 53, 54]. HIF-1 becomes active in reoxygenated regions through the following mechanisms. We reported that the mTOR pathway promotes the synthesis of the HIF-1α protein in glucose- and reoxygenation-dependent manners in irradiated tumors [53]. Moeller *et al*. demonstrated that reoxygenation stabilized the HIF-1α protein and enhanced the translational initiation of HIF-1 targets by increasing ROS levels and stress granule depolymerization, respectively [51]. As a result, activated HIF-1 increases the radioresistance of the tumor vasculature by increasing the amount of the secreted proangiogenic cytokine, VEGF [5, 51, 52, 55]. The involvement of HIF-1 and VEGF has been confirmed in *in vivo* studies; the HIF-1 inhibitor, YC-1, or a neutralizing antibody against VEGF markedly induced apoptosis in endothelial cells and decreased microvessel density after radiation therapy, resulting in radiosensitizing effects in a tumor growth delay assay [51, 52, 54]. Collectively, these findings have provided a rational basis for the combination of radiation and anti-angiogenic therapy in order to enhance the therapeutic effects of radiation.

### Repopulation of radio-surviving cancer cells

Lineage tracking of hypoxic tumor cells *in vivo* revealed the importance of HIF-1 in tumor recurrence after radiation therapy [6]. We constructed a very unique plasmid that expressed Cre recombinase fused to an estrogen receptor (ER^T2^) under the control of a HIF-1-dependent 5HRE promoter, and established a system to tag HIF-1–positive hypoxic tumor cells with luciferase proteins in a tumor xenograft [6]. The resultant lineage tracking revealed that, after surviving radiation therapy, hypoxic tumor cells induced epithelial–mesenchymal transition (EMT) in a HIF-1–dependent manner, translocated toward tumor blood vessels, and eventually caused tumor recurrence after radiation therapy (Fig. 3). The incidence of tumor recurrence was significantly suppressed in tumor-bearing mice treated with the HIF-1 inhibitor, YC-1, and by inhibiting EMT, suggesting a critical role of HIF-1 in tumor repopulation after radiation therapy [6].

## PERSPECTIVES

Accumulating evidence for how HIF-1 becomes active under not only hypoxic conditions but also under normoxic conditions and also for how HIF-1 functions in the induction of radioresistant characteristics in cancer cells and tumor recurrence after radiation therapy have been summarized.

Mathieu *et al*. showed that hypoxia, through the activation of HIFs, induces a hESC-like transcriptional program, including the induced pluripotent stem cell (iPSC) inducers, OCT4, NANOG, SOX2, KLF4, cMYC and microRNA-302 in various cancer cell lines [56]. In addition, it has been suggested that HIF-1 functions in glucose metabolic reprogramming and the cell cycle arrest at the G_0_/G_1_ phase of cancer stem cell–like cells (CSCs) [57]. Moreover, since CSCs have been suggested to exhibit radioresistance through the activation of DNA repair pathways [58], all these findings collectively indicate that HIFs would function in the generation and maintenance of CSCs and are involved in the radioresistance of CSCs.

HIF-1 is now recognized as an excellent molecular target to enhance the therapeutic effects of radiation because of its fundamental roles in tumor radioresistance and post-irradiation tumor recurrence [3–6, 25, 59–61]. However, when attempting to inhibit HIF-1 activity during radiation therapy, it is necessary to consider the timing of combinations because the treatment regimen has been demonstrated to determine whether a HIF-1 inhibitor enhances or inhibits the therapeutic effects of radiation [54]. Our group previously demonstrated that, following a treatment with YC-1 and then radiation, YC-1-mediated increases in tumor hypoxia suppressed the effects of radiation therapy. On the other hand, in a treatment in the reverse order, YC-1 suppressed the post-irradiation upregulation of HIF-1 activity and consequently delayed tumor growth [53, 54]. Based on the combination of HIF-1 blockade with conventional fractionated radiation therapy for cancer patients, the optimization of treatment regimens by performing basic research is needed in order to obtain better therapeutic benefits.

## FUNDING

This review article was prepared under the support of the Funding Program of the Japan Society for the Promotion of Science (JSPS) for NEXT Generation World-Leading Researchers (NEXT Program, No. LS071); of the program of the Japan Science and Technology Agency (JST) for Precursory Research for Embryonic Science and Technology (PRESTO); of the Project of Ministry of Education, Culture, Sports, Science and Technology (MEXT), Japan, for the Development of Innovative Research on Cancer Therapeutics (P-DIRECT); of Grants-in-Aids from MEXT for Scientific Research (B) and for challenging Exploratory Research; and of research grant programs of the Takeda Science Foundation, Relay for Life Japan, Daiichi Sankyo Foundation of Life Science, and Daiwa Securities Health Foundation. Funding to pay the Open Access publication charges for this article was provided by ICRR.
